# Constraint Optimization Model for Dynamic Parking Space Allocation

**DOI:** 10.3390/s24123988

**Published:** 2024-06-19

**Authors:** Abdelrahman Osman Elfaki, Wassim Messoudi, Anas Bushnag, Shakour Abuzneid, Tareq Alhmiedat

**Affiliations:** 1 Faculty of Computers & Information Technology, University of Tabuk, Tabuk 47512, Saudi Arabia; w.messoudi@ut.edu.sa (W.M.); abushnag@ut.edu.sa (A.B.); 2 Department of Cybersecurity and Network, School of Criminal Justice Studies, Roger Williams University, Bristol, RI 02809, USA; sabuzneid@rwu.edu; 3 Artificial Intelligence and Sensing Technologies (AIST) Center, University of Tabuk, Tabuk 47512, Saudi Arabia

**Keywords:** parking management system, dynamic parking space allocation, constraint optimization model

## Abstract

Managing car parking systems is a complex process because multiple constraints must be considered; these include organizational and operational constraints. In this paper, a constraint optimization model for dynamic parking space allocation is introduced. An ad hoc algorithm is proposed, presented, and explained to achieve the goal of our proposed model. This paper makes research contributions by providing an intelligent prioritization mechanism, considering user schedule shifts and parking constraints, and assigning suitable parking slots based on a dynamic distribution. The proposed model is implemented to demonstrate the applicability of our approach. A benchmark is constructed based on well-defined metrics to validate our proposed model and the results achieved.

## 1. Introduction

Finding suitable parking spaces is widely recognized as a significant challenge in densely populated cities. This issue arises primarily because of the limited availability of parking spaces relative to the high number of vehicles on the road [1,2]. By utilizing car parks, the authors of [3,4] proved the significance of using intelligent parking systems to find suitable parking slots. According to ref. [5], parking difficulties arise primarily from inefficiently utilizing existing parking resources. According to ref. [6], searching for parking spaces must become more effective to improve traffic flow.

After years of hard work, many solutions have been proposed to resolve the problems associated with offering suitable parking. Significant developments in the IoT and sensor technology industry have encouraged the advancement of parking systems through sensor applications. However, these applications need to be supported by modern AI techniques [7,8]. Unfortunately, these solutions often lack effective management and fail to optimize the utilization of existing resources at the lowest possible cost. For instance, parking spaces previously reserved for employees in the organization may not be utilized optimally when the employee is on vacation or absent. Designating unique parking slots for visitors is another outcome of a poor parking management system because of the inconsistency in visitor attendance. We often find very crowded parking lots at certain establishments, especially during celebrations, while nearby establishments have empty parking lots. Therefore, it is necessary to implement a flexible parking system that allows parking in one institution to extend to include the empty parking slots of a neighboring institution. Another example of parking mismanagement is the failure to consider employees’ priorities when determining parking arrangements. These priorities could be defined based on proximity to the workplace, accessibility, working shifts, or seniority. The paragraph above highlights the issues addressed in this paper.

This paper develops and presents an ad hoc algorithm representing a constraint optimization model for dynamic parking space allocation. The following three constraints are considered: constraints on user priorities, constraints on parking organization, and constraints on schedule shifts. Hence, the objective function of the proposed model satisfies these three constraints. The proposed model offers an intelligent parking management system with the following features:

1. It provides a suitable parking slot based on dynamic distribution. The distribution is implemented based on working shifts, giving the highest priority to employees of the current working shift.
2. The flexibility of the parking spaces allows for the merging of more than one designated parking slot if needed.
3. Parking reservations enable the system to assign a parking slot for a visitor in advance.
4. A control mechanism reassigns parking slots for absent employees or visitors.
5. A prioritization mechanism is implemented based on predefined criteria.

The proposed model combines these features to create an intelligent, efficient, and adaptable parking management system. Optimization is attained by considering all three constraints as a complete package.

This paper is organized as follows. The Section 2 contains a discussion and analysis of related works, and the strengths and limitations of each work are defined. The Section 3 contains modeling of the proposed model and snapshots of the developed algorithms. First-order logic is used to model the intelligent rules of our proposed model and provide mathematical representations and description models [9,10]. In Section 4, the implementation of our proposed model is presented. A discussion and conclusion are presented in Section 5.

## 2. Related Works

In this section, we discuss related works. Optimization in intelligent parking systems is considered a relatively new area. Therefore, we limited the review to the last five years. We excluded research works focusing on parking challenges related to autonomous vehicles or finding parking slots that specifically include an electric charger, i.e., research works that exclusively target electric vehicles.

The work in [11] developed a constrained optimization model to evaluate the impact of public parking locations on potential traffic congestion, with the optimization goal of minimizing CO_2_ emissions. Prioritization was defined as first come, first serve, and the option of making a reservation was not offered. The authors of [1] developed a multiobjective optimization algorithm for identifying appropriate travel routes and parking lots. Their ad hoc algorithm consisted of the following five weighted factors: the traffic congestion rate, trip distance, availability in the parking lots, parking gate waiting time, and parking cost. Regardless of achieving high accuracy, any previous work we consider needs to include the factors of flexibility and prioritization. The work in [12] proposed a mechanism for shared parking slots based on a prediction system. Their prediction system was developed based on public usage, and it needed more consistency to guarantee accurate results.

In their study, ref. [13] focused on developing an integer linear programming model for the allocation of parking spaces. However, their paper lacks prioritization and validation mechanisms. The research in [14] developed a constraint satisfaction model for allocating suitable parking lots. The authors of [15] developed an ad hoc algorithm based on an equivalent mathematical model to optimize the identification of a suitable parking slot based on parking fees, parking space, search time, and walking time. In [5], a metaheuristic algorithm considering destination location, parking preferences, and time windows was developed to address the dynamic shared parking allocation problem. The previously mentioned works need additional focus on prioritization and feasibility. In [16], the authors proposed a model for identifying suitable parking slots via ant colony optimization. In their model, the researchers considered maximizing the availability of free parking slots as the optimization criterion. This mechanism directed the algorithm to find the best allocation of parking slots that minimize congestion and maximize the number of available vehicle slots.

The work in [17] studied the effects of parking reservation mechanisms on park-and-ride systems. They developed a multimodal agent-based network model to simulate the behavior of park-and-ride users and evaluated various parking reservation strategies. Their objective was to minimize carbon emissions using these strategies. The authors of [18] proposed optimizing real-time parking reservation systems with the use of two objective functions, random arrivals of parking requests, and driver parking unpunctuality. The authors of [19] proposed two shared parking spot allocation models—real-time and fixed-time systems—based on a time window constraint. The branch delimitation algorithm and genetic simulated annealing algorithm were designed to solve the proposed models. In [20], an intelligent parking system was developed using deep learning techniques. The authors employed the YOLOv5 model to detect parked cars. Consequently, parking slots without cars were considered available parking slots. The authors treated the identification of available park slots as an objective function. The authors of [21] designed an intelligent parking system based on the Internet of Things (IoT) and a cloud platform. Finding the nearest available parking slot was an objective of their research.

In [22], an intelligent parking system was developed using an HDDNO-based ML model. The authors forecasted parking lot availability for a given time. The authors of [23] developed an intelligent parking system by utilizing the Markov decision process and a dynamic planning-based reinforcement learning algorithm; the reduction in parking time was an objective function. The authors of [24] designed a system for enhancing the prediction of parking occupancy by utilizing a combination of adaptive neuro-fuzzy inference systems and deep learning techniques. The authors of [25] used ant colony optimization to maximize the net profit of a parking system by defining the most in-demand time. The work in [26] developed an optimization model for an intelligent parking system with an objective function that considered the priority of the demanders. The works in [7] introduced a binary linear programming problem and a genetic-based mat heuristic. The work in [27] demonstrated a deep reinforcement learning-based cooperative approach to address the parking space allocation problem. The work in [28] proposed an ant colony optimization algorithm for parking lot rental to a shared E-scooter service. The work in [29] proposed a low-cost, high-performance middleware solution for unified parking management.

Through an analysis of the works above, it is evident that there are research gaps, which include the following:

- The lack of an intelligent prioritization mechanism that provides better management for parking slots by providing a prioritization system.
- A more accurate prediction system based on parking behavior in public parking spaces might need to be developed because of the varying nature of users. The prediction should be developed based on a diverse group of users with different behaviors.

Table 1 shows a summary of the related works, where each discussed work’s objective function and limitations are presented. The related works are designed to explore the research gaps based on the definition of intelligent parking provided in the Introduction. Hence, the limitations are identified based on these features.

In addition, the related works were classified based on the used technique. Table 2 shows a summary of the techniques used in the related works. This classification paves the way to study the effects of each technique in providing an effective and efficient solution for finding suitable car parking spaces.

## 3. Modeling

As mentioned earlier, our solution aims to optimize the distribution of parking spaces for a given organization, considering constraints such as driver’s priority, scheduled shifts, and parking organization.

**Figure 1:**
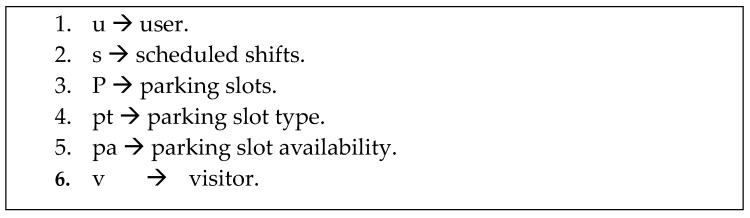
Variables of the proposed model.

**Figure 2:**
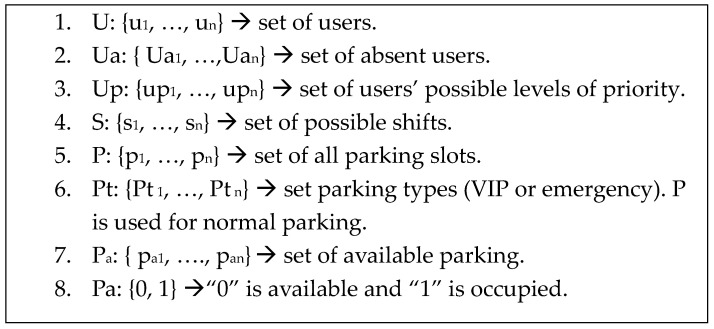
Domain of variables used in our proposed model.

In this section, our proposed model is described as a mathematical model containing three main parts. The first part is a set of variables, the second part is a set of domains, where each variable has a specific domain, and the third part is a set of constraints. An acceptable solution contains variables that are assigned their respective values while considering the constraints. Figure 1 denotes the set of variables in our model, whereas Figure 2 illustrates the set of domains for each variable. The constraints are denoted by Equations (1)–(9). In this work, constraints are described based on three parts as follows:

- User priority: We define a “user” of the parking system as an employee or a visitor who may have the right to park. The priority of an employee is determined by the organization to which they belong. For instance, in hospitals, medical staff have a higher priority than the administrative staff, which means that medical staff deserve the nearest parking slots. In the same context, visitors have a lower priority than employees.
- Scheduled shift: A scheduled shift is defined as a work period during the day. For example, some institutions operate 24 h a day, so they have three work shifts, each with a specific number of hours. The staff assigned to work on the current shift has a higher priority.
- Parking organization: Parking slots could be extended to the neighborhood area (release out). In addition, an organization could rent its parking slots to neighbors if there are available parking slots (lease in). Some slots are reserved for crucial employees or emergencies.

Figure 1 shows the variables that are used in our proposed model. These variables will be used in the designed ad hoc algorithms.

The first variable, “u”, denotes the typical user of a parking space; typically, an employee of an organization owns the parking area. The second variable, “s”, signifies the current working shift. The working shift is a designated time frame for employees to perform their work duties. The third variable, “p”, denotes the parking area owned by a specific organization. In our proposal, this definition is flexible, allowing for the redefinition of multiple parking areas owned by different organizations as single parking areas if necessary. The fourth type, “pt”, represents the type of parking. This type has three possible values including normal, VIP, or emergency. Normal parking refers to regular or standard parking; “VIP” is reserved for higher-level employees. VIP parking should provide convenient and privileged parking for a few individuals. Emergencies offer parking spaces near emergency exits, designated areas for emergency vehicles, or parking spots reserved for emergency personnel. VIP and emergency parking have the highest priorities. The last variable, “v”, indicates a visitor. Visitors need to reserve a parking spot before arriving. Visitor priority is lower than user priority.

Figure 2 depicts the domains containing our proposed variables. We consider the domain as a set and treat it as an array. The first domain indicates the domain of users. The second domain indicates absent users. Declaring a user as absent depends on the organization’s policy. When a user is declared to be absent, their parking will become available. The third domain indicates the priorities of the users. Domain number 4 indicates possible working shifts. Domain number 5 indicates the total number of parking slots. Domain number 6 shows the types of parking slots. Domain number 7 indicates the available parking slots. The last domain contains two values for defining parking assignments from the available parking slots.

The constraints considered in this paper are presented and discussed below.

*∀s ∃t*: *shift*(*s*) ∧ *time*(*t*) ⟹ *∑s* = *t_e_* − *t_s_* (1)

where t_e_ and t_s_ denote the ending and starting times, respectively.

Equation (1) shows that each shift has a specific time slot.

(2) $$\forall p\forall u\forall s\exists t:\mathrm{parking}(p)\;\wedge \;\mathrm{user}(u)\;\wedge \;\mathrm{shift}(s)\;\wedge \;\mathrm{time}(t)\;⟹\;\mathrm{assign}(p,\;u)\wedge \;\sum_{p}\sum_{s}X_{up}^{s}=1\;$$

where $X_{up}^{s}$ shows the user’s parking slot during a specific shift.

Equation (2) states that users must be assigned to one parking slot during a shift.

(3) $$\forall p\forall u\forall s\exists t:\mathrm{parking}(p)\;\wedge \;\mathrm{user}(u)\;\wedge \;\mathrm{shift}(s)\;\wedge \;\mathrm{time}(t)\;⟹\;\sum_{u}X_{up}^{s}\leq 1$$

Each parking slot can only be assigned to one user during a shift.

*∃u_1_ ∃u_2_ ∀s*: *user*(*u_1_*) ∧ *user*(*u_2_*) ∧ *shift*(*s*) ∧ *u_1_∈ s* ∧ *u_2_ ∉ s* ⟹ *priority*(*u_1_*) *> priority*(*u_2_*) (4)

where shift(s) denotes the current shift.

Equation (4) states that a user belonging to a current shift has a higher priority than a user not working during the current shift.

*∀v ∀u*: *visitor*(*v*) ∧ *user*(*u*) ⟹ *priority*(*u*) *> priority*(*v*) (5)

Equation (5) indicates that the priority of a user (organizational employee) is greater than that of a regular visitor.

*∀p ∀u*: *user*(*u*) ∧ *parking*(*p*) ∧ (*priority*(*u*) = *priority*(*p*)) ∨ (*priority*(*u*) *> priority*(*p*)) ⟹ *assign*(*p, u*) (6)

Equation (6) indicates that users are assigned a parking slot of a given type based on their priority level. This priority means that a parking slot could be assigned to a user with the same priority level or higher priority level.

*∀p ∃t*: *parking*(*p*) ∧ *time*(*t*) ∧ *available* (*p, t*) ⟹ *priority*(*p*) − *n* (7)

Equation (7) indicates that if a parking slot remains unoccupied by any user for a predefined time, denoted as “t”, then its priority will decrease by a predefined level, denoted as “n”.

*∀p*: *parking*(*p*) ∧ (*type*(*p, vip*) ∨ *type*(*p, emergency*)) ⟹ *priority*(*p*) = *max* (8)

Equation (8) determines the priority of parking based on the information given. If the parking type is designated explicitly for a VIP, or if it is designated for emergencies, then the priority of that particular parking spot will be set as the maximum priority.

*∀p, ∀u, ∀v* : *parking*(*p*) ∧ *user*(*u*) ∧ *visitor*(*v*) ∧ ((*assign*(*p, u*) ∨ *assign*(*p, v*))
∧ (*absence*(*u*) ∨ *absence*(*v*)) ⟹ *available*(*p*) (9)

Equation (9) indicates that if the parking is assigned to a specific user or a specific visitor and the user or visitor is absent, then this parking becomes available.

Algorithm 1 shows a snapshot of our developed ad hoc dynamic constraint search algorithm.

The proposed ad hoc dynamic constraint search algorithm aims to initialize all the variables and constraints to satisfy the constraint satisfaction theory. The assignment of slots is initialized as an empty list.

Initially, the algorithm checks whether all the variables have been assigned a value. If they have, the search is considered complete; otherwise, it assigns values to all employees and visitors. Employees are assigned first, as they have a higher priority (line 19).
**Algorithm 1**: The proposed ad hoc dynamic constraint search algorithm.1:  Define: 2:   Es **is** all employees 3: Vs **is** all visitors 4:   Ps **is** parking slots 5:   Pa **is** all parking areas 6:   Cs **is** all constraint 7:  **dynamic-search**(assignments, Es, Vs, Ps, Cs) 8:    **if all**(assignments[v] **is not** None **for all** v **in** [Es and Vs]) 9:        **return** assignments//if all variables are assigned, a solution is found 10:   var = **select_unassigned_variable**(Es, Vs, assignments) 11: Ps = **expaned_parking**(Ps) 12:   **for all** slot **in** Ps **do** 13:     **if is_consistent**(assignments, Cs) 14:        result = **csp_algorithm**(assignments, Es, Vs, Ps, Cs) 15:        **if** result **is not** None 16:           **return** result 17:     assignments[var] = None //Backtrack 18:   **return** None //No consistent assignment found **19**: **select_unassigned_variable**(Es, Vs, assignments) **20: for all** employees **in** Es **21:  if** assignments[var] **is** None **22:   return** var **23:  for all** visitor **in** Vs **24: if** assignments[var] **is** None **25:   return** var **26: expaned_parking**(Ps) **27:** if all(p.is_occupied for all p in Ps) **28:**   Ps += Pa.next **29: Return** Ps **30**: **is_consistent**(assignments, Cs): **31: for all** constraint **in** Cs: **32:  if not** constraint(assignments) **33:   return** False **34: return** True

Before we proceed to the parking slots, we check if they are all occupied. If they are, we expand them by adding slots from other parking areas (lines 11 and 26).

After each assignment, we check whether it meets the defined constraints. If it does, we continue; otherwise, we backtrack that assignment and try again with a different slot. Algorithm 2 illustrates how to apply the constraints.

The first part checks whether the user’s shift is the same as the current work shift or whether they are visitors. In both cases, the system rejects the parking assignments. The second part checks whether the user’s priority level does not match the assigned slot. In this case, the system also rejects parking assignments. The third part ensures that another employee does not already occupy the allocated slot. Such constraints can be adjusted based on the institute’s parking policy.
**Algorithm 2:** Applying constraints.1:  **shift_constraint**(assignments) 2:  **for all** an **in** assignments 3:  **if not** a[user].**shift is** shifts.current_shift **or** a[visitor] 4:         **return** False 5:  **return** True 6:  **priority_constraint**(assignments) 7:  **for all** an **in** assignments 8:    **if not** a[user].type **is** parking_slot.type 9:         **return** False 10: **return** True 11: **occupied_constraint**(assignments) 12: **for all** an **in** assignments 13:       **if** a[parking_slot].is_occupied **is** True 14:          **return** False 15:       **else** 16:          a[parking_slot].is_occupied **=** True 17:       **return** True

## 4. Implementation

**Figure 3:**
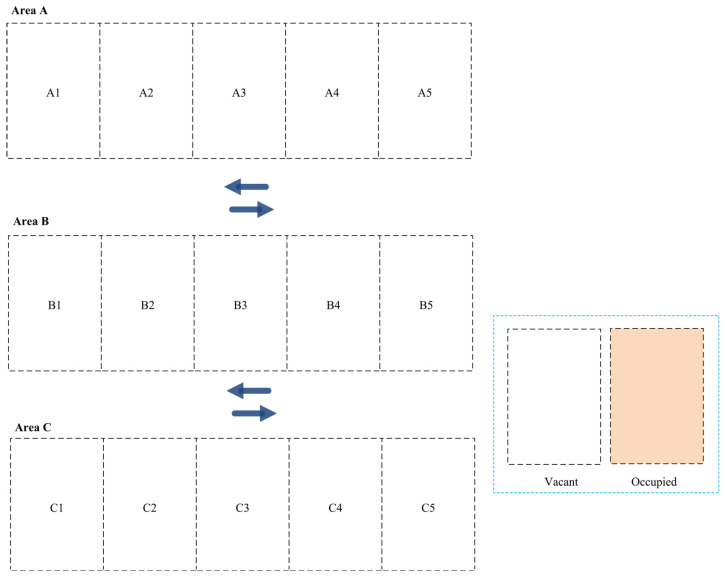
The experimental testbed for the parking area.

This section discusses the experimental testbed we used to assess the applicability and efficiency of the developed dynamic parking space allocation system. Figure 3 presents a parking space consisting of three different areas (A, B, and C), each with five parking slots. Each parking area has its own priority, which can be set before the distribution process. In our experiments, we consider Area A to have the highest priority, Area B to have a medium priority, and Area C to have the lowest priority.

We considered two scenarios for validation, each consisting of several employees and visitors who require a parking slot. Table 2 presents the first scenario’s number of employees and visitors along with their shifts and priorities. In this scenario, there are two different types of drivers (employees and visitors). In addition, there are three different shits (A, B, and C), and each driver has a priority, which are high priority (H. priority), medium priority (M. priority), and low priority (L. priority), as presented in Table 3.

For instance, Shift A requires six parking slots for employees and seven for visitors. Among the six employees, four had high priority, one had medium priority, and one had low priority, whereas among the seven visitors, two had high priority, three had medium priority, and two had low priority. The same is true for Shift B, where there are 10 parking slots for employees and 4 for visitors. Finally, for Shift C, 11 parking slots are required for employees, and 2 parking slots are required for visitors.

**Figure 4:**
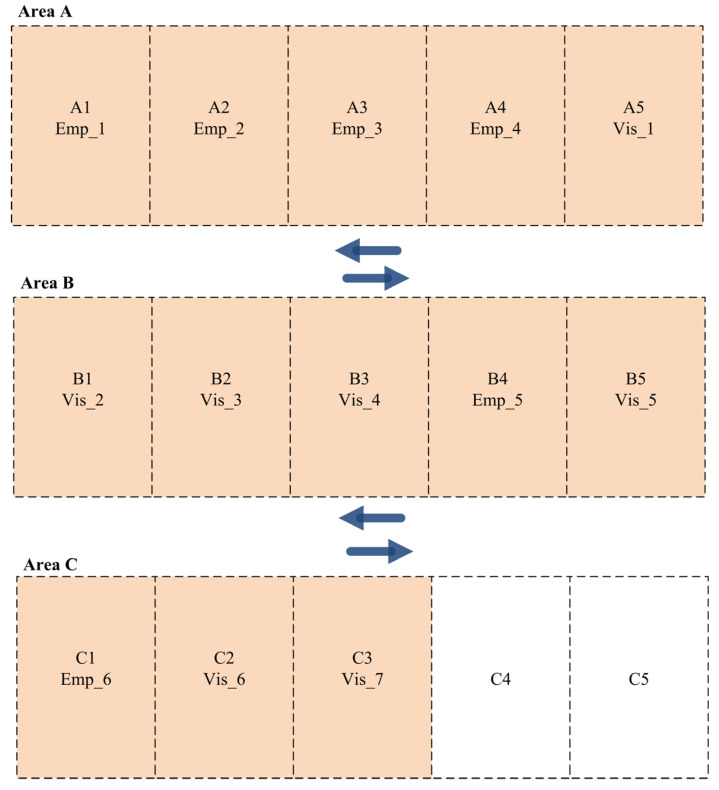
Parking slot allocation among employees and visitors for Scenario 1 (Shift A).

The results of the parking slot allocation among employees and visitors for the three shifts are obtained from the new dynamic parking space allocation function. For instance, the parking slots are distributed among the employees and visitors in Shift A with the best distribution based on their priorities, as presented in Figure 4, where the employees and visitors with the highest priority are allocated parking slots in Area A. Consequently, the medium- and lowest-priority drivers are assigned parking slots in Areas B and C.

**Figure 5:**
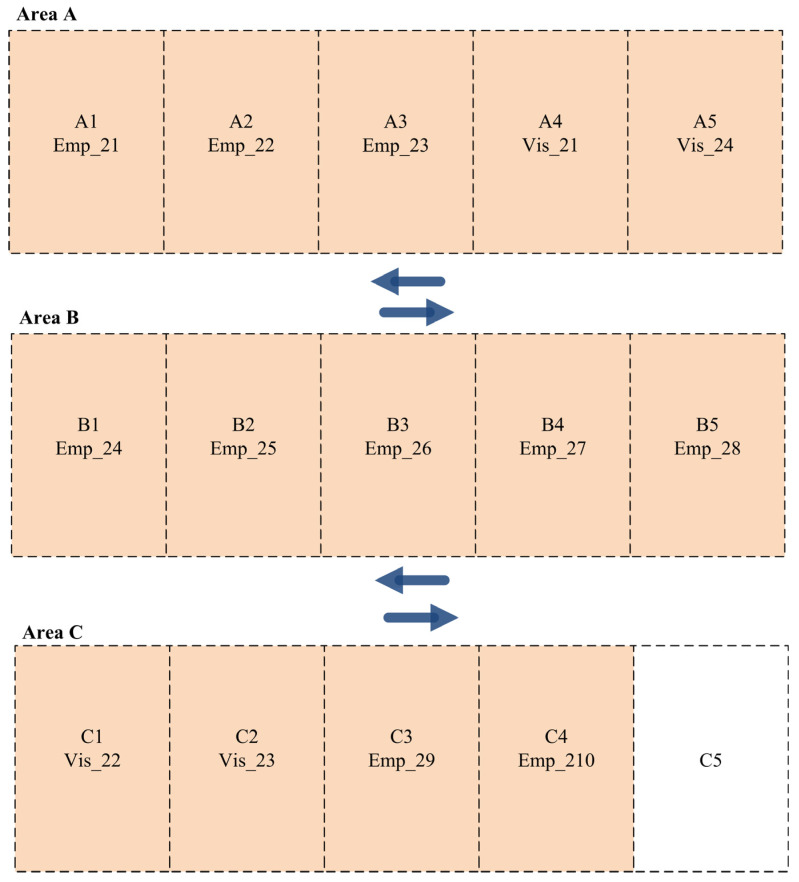
Parking slot allocation for employees and visitors in Scenario 1 (Shift B).

On the other hand, the employees and visitors in Shift B are distributed among the available parking slots based on their priorities. Figure 5 presents the best parking slot allocation among employees and visitors for Shift B. As presented in the figure below, three employees and two visitors are allocated to parking slots in Area A. In contrast, medium-priority drivers are assigned to parking slots in Area B. Finally, drivers with the lowest priorities are allocated to parking slots in Area C.

**Figure 6:**
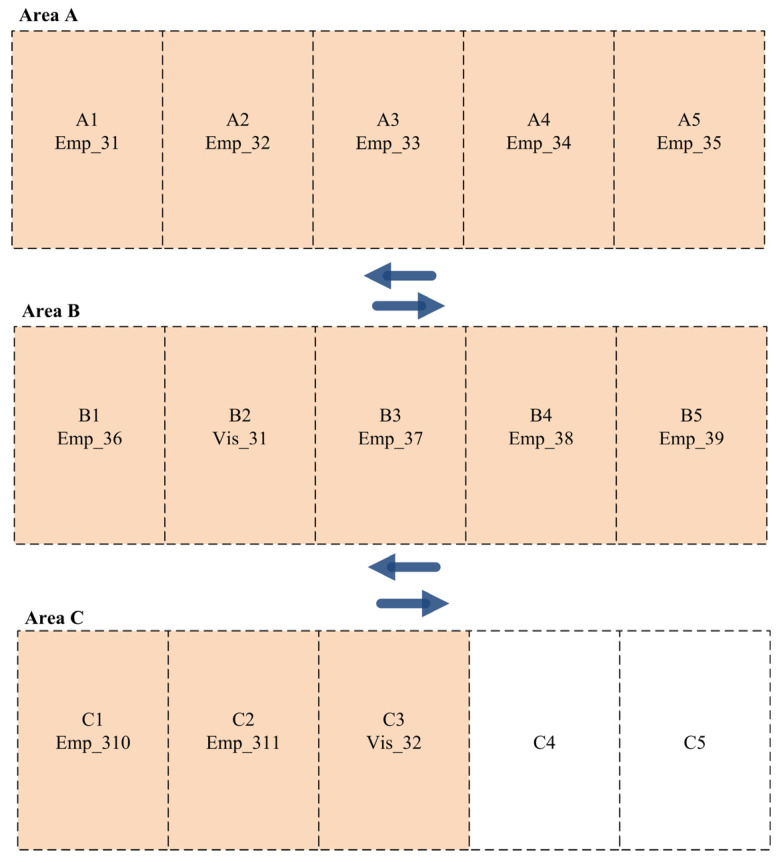
Parking slot allocation for employees and visitors in Scenario 1 (Shift C).

The parking slot allocation for employees and visitors in Scenario 1 (Shift C) is presented in Figure 6. Employees occupy Area A based on their high priority, and employees and visitors with lower priorities are allocated spaces in Areas B and C.

The second scenario is presented in Table 4, where we focused on the medium and low priorities for the employees and visitors to validate the efficiency of the developed dynamic parking space allocation function. For instance, Shift A has four employees (1: H. Priority, 2 M. Priority, and 1 L. Priority) and 10 visitors (3 H. Priority, 1 M. Priority, and 6 L. Priority).

**Figure 7:**
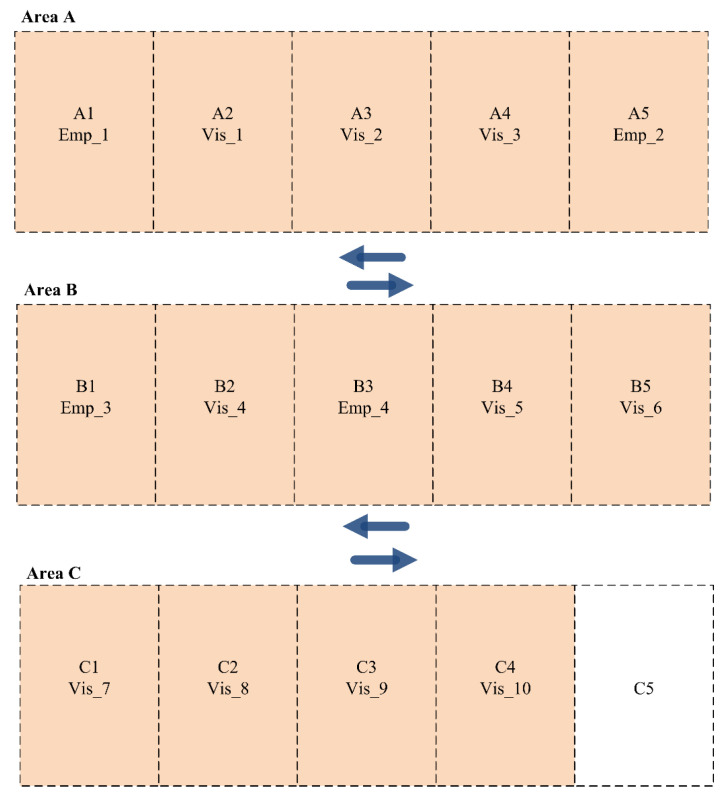
Parking slot allocation for employees and visitors in Scenario 2 (Shift A).

The obtained results of the developed dynamic parking space allocation system are presented in Figure 7 for Scenario 2 (Shift A). As presented, employees and visitors with high priorities are allocated to parking slots in Area A. In contrast, other lower-priority employees and visitors are allocated to parking slots in Areas B and C.

**Figure 8:**
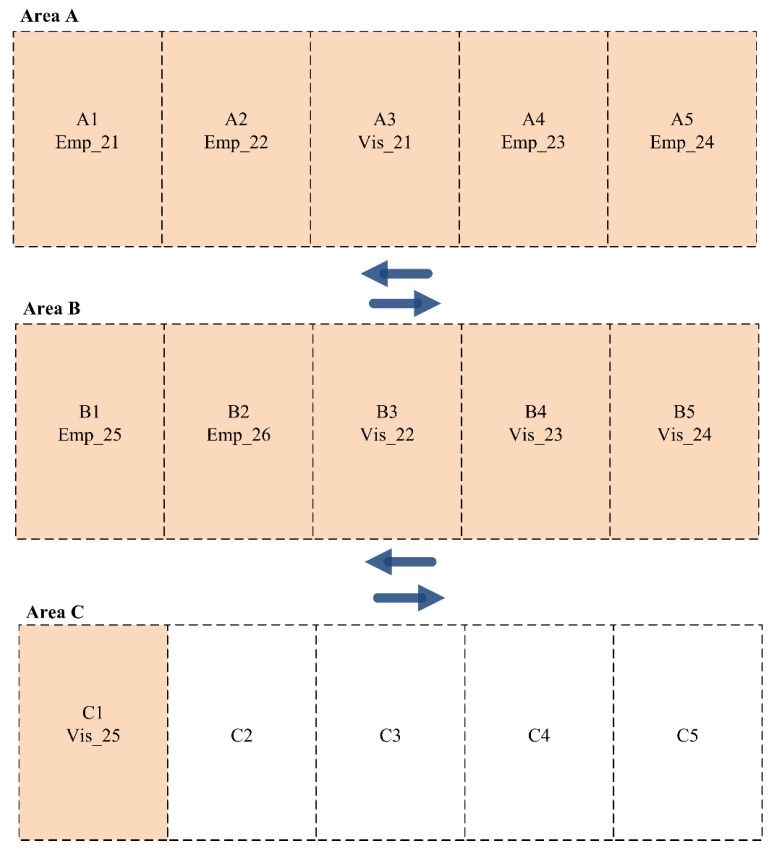
Parking slot allocation among employees and visitors for Scenario 2 (Shift B).

The second shift (Shift B) involved six employees (2 H. Priority and 4 L. Priority) and five visitors (1 M. Priority and 4 L. Priority). Employees and visitors with high priorities are allocated to parking slots in Area A, whereas others are allocated to parking slots in Areas B and C, as presented in Figure 8.

**Figure 9:**
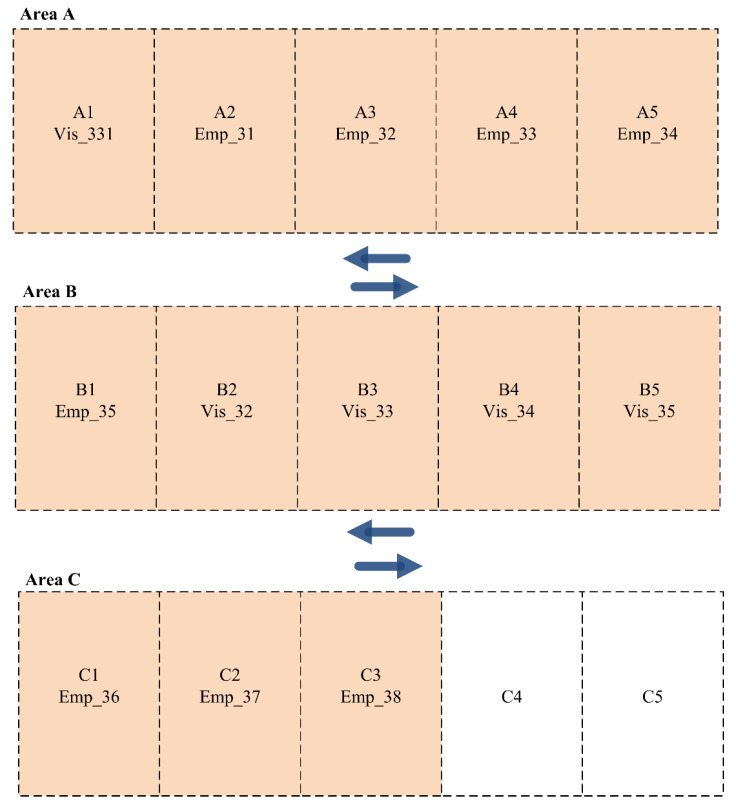
Parking slot allocation among employees and visitors for Scenario 2 (Shift C).

Finally, Shift C consists of eight employees (5 M. Priority and 8 L. Priority) and 5 visitors (1 H. Priority and 4 L. Priority). As noted in this scenario, a single visitor has high priority. Therefore, the developed dynamic parking space allocation function allocates a single visitor with H. Priority to Area A. In contrast, other employees with M. Priority are allocated to Area A. However, other employees and visitors are allocated to Areas B and C, as presented in Figure 9.

For more validation results, we considered a new practical experiment where a car parking dataset (https://data.world/datagov-uk/14061134-f8c0-4e72-83bd-d3216d36f82f/workspace/project-summary?agentid=datagov-uk&datasetid=14061134-f8c0-4e72-83bd-d3216d36f82f, accessed on 2 June 2024) was adopted. The new distributions of employees and visitors are depicted in Table 5.

**Figure 10:**
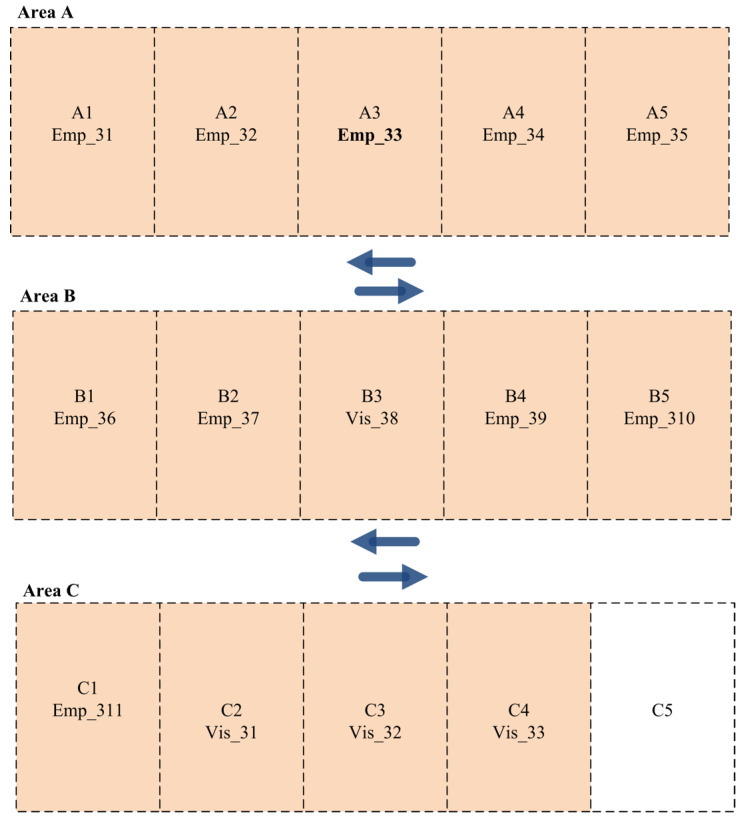
Parking slot allocation among employees and visitors for Scenario 3.

After considering the above scenario, the obtained results from the developed parking management system are presented in Figure 10.

## 5. Discussion and Conclusions

Our proposed constraint optimization model for dynamic parking space allocation is validated using ad hoc algorithms, where all the variables are assigned values from their domains while considering the associated constraints. Along with their implementation in Section 4, Algorithms 1 and 2 demonstrate the correctness and applicability of our proposed model. The remaining contributions generated from our proposed model are demonstrated by utilizing a benchmark to compare our work with related studies.

In this section, a benchmark consisting of seven parameters is used to evaluate the proposed model. This benchmark was developed by analyzing related works. The benchmark parameters are defined below. The aim of this benchmark is to shed light on our contributions by comparing our findings with those of related studies.

1. Dynamic distribution means there are no fixed allocated parking spaces for specific cars or individuals. Dynamic allocations are based on a dynamic objective function. For instance, the dynamic objective function could prioritize job responsibilities, health conditions, or high-ranking visitors.
2. Flexible parking spaces mean that another organization can utilize the parking spaces of a particular organization. Therefore, parking slots are defined based on real-time demand.
3. The average waiting time is the average time a vehicle spends waiting to find an available parking space or waiting in line to enter or exit a parking facility.
4. The average distance to reach the appropriate slot is a metric used to evaluate the efficiency and convenience of a parking facility. This refers to the average distance a vehicle needs to travel from the entrance or a designated point to reach a suitable parking slot.
5. Confusion refers to the number of incorrect slot assignments. It refers to the frequency or counts of instances where a vehicle is assigned to an incorrect parking slot.
6. Time to full capacity utilization means measuring the time for full-slot assignments. It refers to the duration it takes for all the available slots in a parking facility to be fully occupied or assigned. It measures the time from starting slot assignments until all the slots are utilized.
7. Priority assignment measures the average of assigned slots with a specified priority. This refers to the process of assigning parking slots based on defined priorities. The metric associated with priority assignment is the average of the assigned slots with the specified priority.

Table 6 summarizes these benchmark parameter descriptions, calculation equations, objective functions, and cited references.

In Section 4, we explain how our proposed model satisfies these seven parameters. The obtained results prove that the proposed system has dynamic allocation feature capabilities since the parking slots were distributed among employees and visitors based on their priorities, with no fixed parking slots for anyone. On the other hand, the system can provide flexible parking spaces, which means that free spaces are distributed to employees and visitors regardless of their organization type (identity).

In addition, the system achieves the minimum average waiting time and confusion rate, as employees and visitors receive parking slot IDs before visiting the organization. Moreover, the system attains the best priority assignment rate since the parking slots are divided among employees and visitors based on their shifts and priorities. To validate our findings, Table 7 summarizes the comparison of our proposed model with the selected works from related studies; Table 5 shows the benchmark results.

## Figures and Tables

**Table 1:** Summary of related works.

Work	Objective Function	Limitation
[11]	Minimizing CO_2_ emissions by reducing searching time	Prioritization is defined as first come, first serve, which denies the option of reservation
[1]	Finding appropriate travel routes	Lacks the factors of flexibility and prioritization
[12]	Predicting available parking slot	Their prediction system was developed based on public usage and lacks flexibility
[13]	Allocation of parking spaces	Lacks prioritization mechanism
[14]	Allocating a suitable parking lot	Lacks prioritization mechanism
[15]	Allocating a suitable parking lot	Lacks prioritization mechanism
[5]	Consider parking preferences	Lacks prioritization and flexibility mechanisms
[16]	Maximizing the availability of free parking	Lacks prioritization and flexibility mechanisms
[17]	Minimizing carbon emissions by simulating the behavior of park-and-ride users	Lacks prioritization and flexibility mechanisms
[18]	Organizing random arrivals of parking requests	Lacks prioritization and flexibility mechanisms
[19]	Finding a parking slot based on time window constraint	Lacks prioritization and flexibility mechanisms
[20]	Finding available park slots	Lacks prioritization and flexibility mechanisms
[21]	Finding the nearest available parking slot	Lacks prioritization and flexibility mechanisms
[22]	Forecasting parking lot availability	Lacks prioritization and flexibility mechanisms
[23]	Reduction in parking time	Lacks prioritization
[24]	Prediction of parking occupancy	Lacks prioritization and flexibility mechanisms
[25]	Defining the most demanded time	Lacks prioritization and flexibility mechanisms
[26]	Considering the priority of the demanders	Lacks flexibility mechanism
[7]	Distribution of parking slots satisfying the constraints	Lacks prioritization and flexibility mechanisms
[27]	Cost-based method of parking allocation	Lacks prioritization
[28]	Reducing wild parking habits	Lacks prioritization and flexibility mechanisms
[29]	Enhancement in the usability of the parking system	Lacks prioritization and flexibility mechanisms

**Table 2:** Summary of the techniques used in the related works.

Work	Technique
[5]	Tabu search algorithm
[7,11,13,17,19,26]	Genetic algorithm
[1,14,15,29]	Ad hoc algorithms
[12]	Recurrent neural network algorithm
[16,25,28]	Ant colony optimization
[18]	Stochastic optimization
[20,21,22,24]	Deep learning
[23,27]	Reinforcement learning

**Table 3:** The distributions of employees and visitors in Scenario 1 among three shifts according to their priorities.

Type	Shift and Numbers	H. Priority	M. Priority	L. Priority
Employee	A: 6	4	1	1
	B: 10	3	5	2
	C: 11	6	3	2
Visitor	A: 7	2	3	2
	B: 4	1	2	1
	C: 2	1	0	1

**Table 4:** The distributions of the employees and visitors in Scenario 2 in 3 shifts based on their priorities.

Type	Shift and Numbers	H. Priority	M. Priority	L. Priority
Employee	A: 4	1	2	1
	B: 6	2	0	4
	C: 8	0	5	3
Visitor	A: 10	3	1	6
	B: 5	0	1	4
	C: 5	1	4	0

**Table 5:** The distributions of employees and visitors in Scenario 3 according to their priorities.

Type	Shift and Numbers	H. Priority	M. Priority	L. Priority
Employee	A: 11	9	2	0
Visitor	A: 3	0	1	2

**Table 6:** Description of the benchmark.

Parameter	Description	Calculation	Objective	Reference
Dynamic allocation	There are no fixed allocated parking spaces for specific cars or individuals.	Da = ¬(p,u)	Maximize Da	[13]
Flexible parking space	The parking space of a particular organization can be utilized by another organization at a different time.	Ps = {p_1_, p_2_,…,p_n_}	Activate Ps	[16]
Average waiting time	This parameter measures the average time to reach the appropriate slot.	$W_{t}={(t}_{p}-t_{q})$ ${Avg(W}_{t})=\frac{\sum {(W}_{t})}{Np}$	Minimize W_t_	[19]
Average distance	This parameter measures the average distance to reach the appropriate slot.	$D_{t}={(D}_{p}-D_{q})$ ${Avg(D}_{t})=\frac{\sum {(D}_{t})}{Np}$	Minimize D_t_	[11]
Confusion	This parameter measures the number of incorrect slot assignments.	${Avg(C}_{t})=\frac{\sum {(I}_{s})}{Np}$	Minimize C_t_	[30]
Time to full capacity utilization	This parameter measures the time for filling all the slot assignments.	Ft = t − t_Full_assign_	Maximize Ft	[26]
Priority assignment	This parameter measures the average of assigned slots with the specified priority.	Pr_a_ = $\frac{\sum {(A}_{s})}{pr}$	Maximize Pr_a_	[23]

Da: dynamic allocation; Ps: parking space; U: user; p: park; q: queue; Np: number of packed slots in a specific time; Is: incorrect assignment; Dt: average distance; tFull_assign: time of full slots assignments; As: assigned slots; Pra: priority assignment; Pr: priority.

**Table 7:** Benchmark results.

Work	Dynamic Allocation	Flexible Parking Space	Average Waiting Time	Average Distance	Confusion	Time to Full Capacity Utilization	Priority Assignment
[11]	x	x	√	x	x	√	x
[1]	√	x	√	x	x	√	x
[12]	√	x	√	√	x	√	x
[13]	√	x	√	x	x	√	x
[14]	x	x	√	√	x	√	x
[15]	√	x	√	x	√	√	x
[5]	√	x	√	x	√	√	x
[16]	x	x	√	x	x	x	x
[17]	√	x	√	√		x	x
[18]	x	x	x	x	√	x	x
[19]	√	x	x	√	√	x	x
[20]	x	x	x	√	√	x	x
[21]	x	x	x	√	√	x	x
[22]	√	x	x	√	√	x	x
[23]	√	x	x	√	√	x	x
[24]	√	x	√	√	√	x	x
[25]	√	x	√	√	√	x	x
[26]	√	x	√	√	√	x	x
[7]	√	x	x	x	√	√	x
[27]	√	√	x	x	x	x	x
[28]	√	x	x	x	x	x	x
[29]	√	x	x	x	x	x	x
The proposed model	√	√	√	√	√	√	√

## Data Availability

Data are contained within the article.
